# Supplementary material for: Connectome‐based predictive modeling of brain pathology and cognition in autosomal dominant Alzheimer's disease
Source: Alzheimers Dement. 2025 Mar 20;21(3):e70061. doi: 10.1002/alz.70061 (PMC11923559; doi:10.1002/alz.70061)
Supplement: Supplementary file 1 — Supporting information [file ALZ-21-e70061-s002.docx]

**Supplementary Results**

**CPM Modeling for Non-carriers**

CPM modeling for non-carriers were non-significant (Figure S1).

**
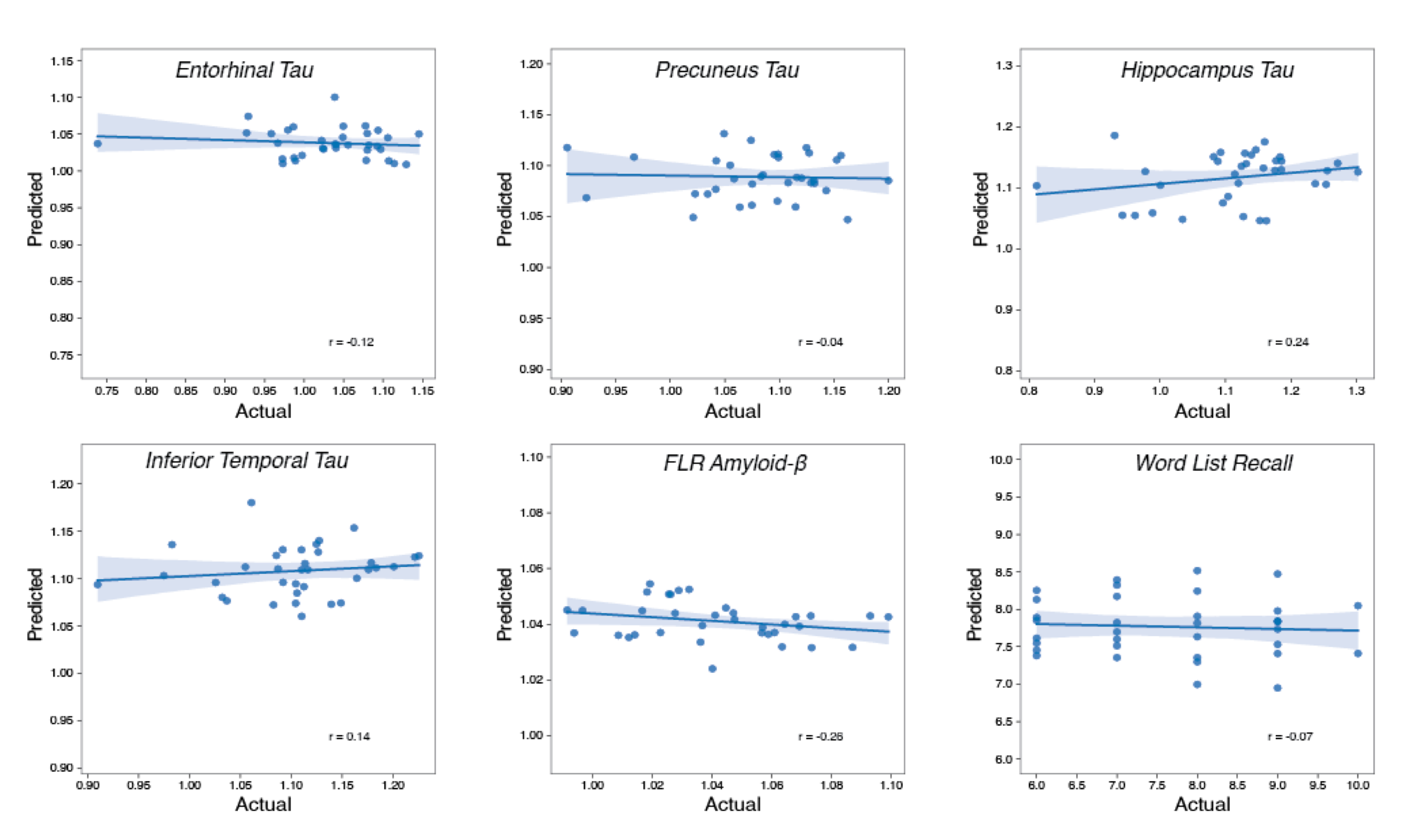
**

*Figure S1: Connectome-based Predictive Modeling in PSEN1 non-carriers: We applied the CPM modeling between FC across parcels and the tau concentrations in the entorhinal, precuneus, hippocampus and IT. The amyloid-β in FLR cortex and word list recall total cognitive scores in the non-carriers within the COLBOS datasets were also investigated. We find that the model is weakly predictive for hippocampal values but not any others.*

**CPM Modeling without outliers**

Removing the two outliers with most high motion frames (>0.2mm), we obtained significant predictions for tau concentrations across entorhinal cortex (Positive network: r(28) = 0.40, p < 0.01; Negative network: r(28) = 0.53, p < 0.001), precuneus (Positive network: r(28) = 0.30, p = 0.02; Negative network: r(28) = 0.48, p < 0.001) and IT (Positive network: r(28) = 0.24, p < 0.05; Negative network: r(28) = 0.46, p < 0.001). Hippocampal tau (Positive network: r(28) = 0.22, p = 0.052; Negative network: r(28) = 0.28, p = 0.035) and amyloid-β in FLR (Positive network: r(28) = 0.06, p = 0.195; Negative network: r(28) = 0.29, p = 0.03) were significant for negative network but not for positive. Word list recall cognitive score prediction was significant for the positive network (Positive network: r(28) = 0.37, p=0.008; Negative network: r(28) = 0.17, p=0.18).

**Impact of association thresholds on prediction accuracies**

*Table S1: Prediction Accuracy of CPM model for different thresholds of association between brain FC and tau measures.*

|  | **Prediction Accuracy** *mean (std)* | | | | | | | |
| --- | --- | --- | --- | --- | --- | --- | --- | --- |
|  | ***Positive Network*** | | | | ***Negative Network*** | | | |
|  | 0.2 | 0.1 | 0.05 | 0.01 | 0.2 | 0.1 | 0.05 | 0.01 |
| Entorhinal TAU | 0.49 (0.04) | 0.49 (0.05) | 0.48 (0.04) | 0.47 (0.04) | 0.53 (0.03) | 0.54 (0.04) | 0.54 (0.03) | 0.54 (0.03) |
| Precuneus TAU | 0.35 (0.06) | 0.33 (0.06) | 0.32 (0.05) | 0.30 (0.05) | 0.47 (0.05) | 0.47 (0.05) | 0.46 (0.05) | 0.45 (0.05) |
| Hippocampus TAU | 0.31 (0.05) | 0.33 (0.06) | 0.31 (0.06) | 0.32 (0.05) | 0.37 (0.04) | 0.38 (0.04) | 0.36 (0.05) | 0.36 (0.04) |
| InferiorTemporal TAU | 0.34 (0.06) | 0.32 (0.06) | 0.29 (0.06) | 0.28 (0.05) | 0.45 (0.05) | 0.46 (0.06) | 0.46 (0.05) | 0.46 (0.05) |
| Amyloid FLR | 0.17 (0.06) | 0.15 (0.07) | 0.12 (0.07) | 0.09 (0.07) | 0.27 (0.04) | 0.29 (0.04) | 0.29 (0.04) | 0.28 (0.05) |
| Word List Recall | 0.37 (0.06) | 0.38 (0.04) | 0.38 (0.05) | 0.39 (0.05) | 0.34 (0.08) | 0.27 (0.07) | 0.25 (0.07) | 0.23 (0.08) |

**Supplementary Methods**

The following text is generated from the fMRIPrep pipeline intended to be used verbatim (CC0) license to allow for clear, consistent reproduction of preprocessing steps (<https://fmriprep.org/en/20.2.0/citing.html>).

***Anatomical data preprocessing***

The T1-weighted (T1w) image was corrected for intensity non-uniformity (INU) with `N4BiasFieldCorrection`, distributed with ANTs 2.3., and used as T1w-reference throughout the workflow.

The T1w-reference was then skull-stripped with a *Nipype* implementation of the `antsBrainExtraction.sh` workflow (from ANTs), using OASIS30ANTs as target template.

Brain tissue segmentation of cerebrospinal fluid (CSF), white-matter (WM) and gray-matter (GM) was performed on the brain-extracted T1w using `fast` [FSL 6.0.1, @fsl_fast].

Brain surfaces were reconstructed using `recon-all` [FreeSurfer 6.0.0, , @fs_reconall], and the brain mask estimated previously was refined with a custom variation of the method to reconcile ANTs-derived and FreeSurfer-derived segmentations of the cortical gray-matter of Mindboggle [@mindboggle].

Volume-based spatial normalization to one standard space (MNI152NLin2009cAsym) was performed through nonlinear registration with `antsRegistration` (ANTs 2.3.1), using brain-extracted versions of both T1w reference and the T1w template.

The following template was selected for spatial normalization:

*ICBM 152 Nonlinear Asymmetrical template version 2009c* [@mni152nlin2009casym, MNI152NLin2009cAsym].

***Functional data preprocessing***

For each of the 2 BOLD runs found per subject (across all tasks and sessions), the following preprocessing was performed. First, a reference volume and its skull-stripped version were generated using a custom methodology of fMRIPrep. The BOLD reference was then co-registered to the T1w reference using `bbregister` (FreeSurfer) which implements boundary-based registration [@bbr]. Co-registration was configured with nine degrees of freedom to account for distortions remaining in the BOLD reference.

Head-motion parameters with respect to the BOLD reference (transformation matrices, and six corresponding rotation and translation parameters) are estimated before any spatiotemporal filtering using `mcflirt` [FSL 6.0.1, @mcflirt]. BOLD runs were slice-time corrected using `3dTshift` from AFNI 20190100 [@afni].

The BOLD time-series, were resampled to surfaces on the following spaces: fsaverage6. The BOLD time-series (including slice-timing correction when applied) were resampled onto their original, native space by applying a single, composite transform to correct for head-motion and susceptibility distortions. These resampled BOLD time-series will be referred to as “preprocessed BOLD in original space”, or just “preprocessed BOLD”.

The BOLD time-series were resampled into standard space, generating a “preprocessed BOLD run in ['MNI152NLin2009cAsym'] space”. First, a reference volume and its skull-stripped version were generated using a custom methodology of fMRIPrep.

Several confounding time-series were calculated based on the preprocessed BOLD: framewise displacement (FD), DVARS and three region-wise global signals. FD and DVARS are calculated for each functional run, both using their implementations in Nipype [following the definitions by Power et al., 2013]. The three global signals are extracted within the CSF, the WM, and the whole-brain masks.

Additionally, a set of physiological regressors were extracted to allow for component-based noise correction [CompCor, Behzadi et al., 2007]. Principal components are estimated after high-pass filtering the preprocessed BOLD time-series (using a discrete cosine filter with 128s cut-off) for the two CompCor variants: temporal (tCompCor) and anatomical (aCompCor). tCompCor components are then calculated from the top 5% variable voxels within a mask covering the subcortical regions. This subcortical mask is obtained by heavily eroding the brain mask, which ensures it does not include cortical GM regions.

For aCompCor, components are calculated within the intersection of the aforementioned mask and the union of CSF and WM masks calculated in T1w space, after their projection to the native space of each functional run (using the inverse BOLD-to-T1w transformation). Components are also calculated separately within the WM and CSF masks. For each CompCor decomposition, the k components with the largest singular values are retained, such that the retained components' time series are sufficient to explain 50 percent of variance across the nuisance mask (CSF, WM, combined, or temporal). The remaining components are dropped from consideration.

The head-motion estimates calculated in the correction step were also placed within the corresponding confounds file. The confound time series derived from head motion estimates and global signals were expanded with the inclusion of temporal derivatives and quadratic terms for each (Satterthwaite et al., 2013). Frames that exceeded a threshold of 0.2 mm FD were annotated as motion outliers.

All resamplings can be performed with a single interpolation step by composing all the pertinent transformations (i.e. head-motion transform matrices, susceptibility distortion correction when available, and co-registrations to anatomical and output spaces).

Gridded (volumetric) resamplings were performed using `antsApplyTransforms` (ANTs), configured with Lanczos interpolation to minimize the smoothing effects of other kernels [@lanczos]. Non-gridded (surface) resamplings were performed using `mri_vol2surf`

(FreeSurfer).

**References:**

1. Esteban O, Markiewicz CJ, Blair RW, Moodie CA, Isik AI, Erramuzpe A, Kent JD, Goncalves M, DuPre E, Snyder M, Oya H, Ghosh SS, Wright J, Durnez J, Poldrack RA, Gorgolewski KJ. fMRIPrep: a robust preprocessing pipeline for functional MRI. Nat Meth. 2018; doi:[**10.1038/s41592-018-0235-4**](https://doi.org/10.1038/s41592-018-0235-4)

2. fMRIPrep Available from: <https://fmriprep.org/en/20.2.0/citing.html>

3. Gorgolewski K, Burns CD, Madison C, Clark D, Halchenko YO, Waskom ML, Ghosh SS. Nipype: a flexible, lightweight and extensible neuroimaging data processing framework in python. Front Neuroinform. 2011 Aug 22;5(August):13. doi:[**10.3389/fninf.2011.00013**](https://doi.org/10.3389/fninf.2011.00013).

4. Gorgolewski KJ, Esteban O, Ellis DG, Notter MP, Ziegler E, Johnson H, Hamalainen C, Yvernault B, Burns C, Manhães-Savio A, Jarecka D, Markiewicz CJ, Salo T, Clark D, Waskom M, Wong J, Modat M, Dewey BE, Clark MG, Dayan M, Loney F, Madison C, Gramfort A, Keshavan A, Berleant S, Pinsard B, Goncalves M, Clark D, Cipollini B, Varoquaux G, Wassermann D, Rokem A, Halchenko YO, Forbes J, Moloney B, Malone IB, Hanke M, Mordom D, Buchanan C, Pauli WM, Huntenburg JM, Horea C, Schwartz Y, Tungaraza R, Iqbal S, Kleesiek J, Sikka S, Frohlich C, Kent J, Perez-Guevara M, Watanabe A, Welch D, Cumba C, Ginsburg D, Eshaghi A, Kastman E, Bougacha S, Blair R, Acland B, Gillman A, Schaefer A, Nichols BN, Giavasis S, Erickson D, Correa C, Ghayoor A, Küttner R, Haselgrove C, Zhou D, Craddock RC, Haehn D, Lampe L, Millman J, Lai J, Renfro M, Liu S, Stadler J, Glatard T, Kahn AE, Kong X-Z, Triplett W, Park A, McDermottroe C, Hallquist M, Poldrack R, Perkins LN, Noel M, Gerhard S, Salvatore J, Mertz F, Broderick W, Inati S, Hinds O, Brett M, Durnez J, Tambini A, Rothmei S, Andberg SK, Cooper G, Marina A, Mattfeld A, Urchs S, Sharp P, Matsubara K, Geisler D, Cheung B, Floren A, Nickson T, Pannetier N, Weinstein A, Dubois M, Arias J, Tarbert C, Schlamp K, Jordan K, Liem F, Saase V, Harms R, Khanuja R, Podranski K, Flandin G, Papadopoulos Orfanos D, Schwabacher I, McNamee D, Falkiewicz M, Pellman J, Linkersdörfer J, Varada J, Pérez-García F, Davison A, Shachnev D, Ghosh S. Nipype: a flexible, lightweight and extensible neuroimaging data processing framework in Python. 2017. doi:[**10.5281/zenodo.581704**](https://doi.org/10.5281/zenodo.581704).

5. Tustison NJ, Avants BB, Cook PA, Zheng Y, Egan A, Yushkevich PA, Gee JC. N4ITK: improved N3 bias correction. IEEE Trans Med Imaging. 2010 Jun;29(6):1310–20. doi:[**10.1109/TMI.2010.2046908**](https://doi.org/10.1109/TMI.2010.2046908).

6. Dale A, Fischl B, Sereno MI. Cortical Surface-Based Analysis: I. Segmentation and Surface Reconstruction. Neuroimage. 1999;9(2):179–94. doi:[**10.1006/nimg.1998.0395**](https://doi.org/10.1006/nimg.1998.0395).

7. Fonov VS, Evans AC, McKinstry RC, Almli CR, Collins DL. Unbiased nonlinear average age-appropriate brain templates from birth to adulthood. NeuroImage; Amsterdam. 2009 Jul 1;47:S102. doi:[**10.1016/S1053-8119(09)70884-5**](https://doi.org/10.1016/S1053-8119(09)70884-5).

8. Avants BB, Epstein CL, Grossman M, Gee JC. Symmetric diffeomorphic image registration with cross-correlation: evaluating automated labeling of elderly and neurodegenerative brain. Med Image Anal. 2008 Feb;12(1):26–41. doi:[**10.1016/j.media.2007.06.004**](https://doi.org/10.1016/j.media.2007.06.004).

9. Jenkinson M, Bannister P, Brady M, Smith S. Improved optimization for the robust and accurate linear registration and motion correction of brain images. Neuroimage. 2002 Oct;17(2):825–41. doi:[**10.1006/nimg.2002.1132**](https://doi.org/10.1006/nimg.2002.1132).

10. Andersson JLR, Skare S, Ashburner J. How to correct susceptibility distortions in spin-echo echo-planar images: application to diffusion tensor imaging. Neuroimage. 2003 Oct;20(2):870–88. doi:[**10.1016/S1053-8119(03)00336-7**](https://doi.org/10.1016/S1053-8119(03)00336-7).

11. Cox RW. AFNI: software for analysis and visualization of functional magnetic resonance neuroimages. Comput Biomed Res. 1996 Jun;29(3):162–73. doi:[**10.1006/cbmr.1996.0014**](https://doi.org/10.1006/cbmr.1996.0014).

12. Jenkinson M. Fast, automated, N-dimensional phase-unwrapping algorithm. Magn Reson Med. 2003 Jan;49(1):193–7. doi:[**10.1002/mrm.10354**](https://doi.org/10.1002/mrm.10354).

13. Huntenburg JM. Evaluating nonlinear coregistration of BOLD EPI and T1w images. Freie Universität Berlin; 2014. Available from: [**http://hdl.handle.net/11858/00-001M-0000-002B-1CB5-A**](http://hdl.handle.net/11858/00-001M-0000-002B-1CB5-A).

14. Wang S, Peterson DJ, Gatenby JC, Li W, Grabowski TJ, Madhyastha TM. Evaluation of Field Map and Nonlinear Registration Methods for Correction of Susceptibility Artifacts in Diffusion MRI. Front Neuroinform. 2017 [cited 2017 Feb 21];11. doi:[**10.3389/fninf.2017.00017**](https://doi.org/10.3389/fninf.2017.00017).

15. Treiber JM, White NS, Steed TC, Bartsch H, Holland D, Farid N, McDonald CR, Carter BS, Dale AM, Chen CC. Characterization and Correction of Geometric Distortions in 814 Diffusion Weighted Images. PLoS One. 2016 Mar 30;11(3):e0152472. doi:[**10.1371/journal.pone.0152472**](https://doi.org/10.1371/journal.pone.0152472).

16. Greve DN, Fischl B. Accurate and robust brain image alignment using boundary-based registration. Neuroimage. 2009 Oct;48(1):63–72. doi:[**10.1016/j.neuroimage.2009.06.060**](https://doi.org/10.1016/j.neuroimage.2009.06.060).

17. Zhang Y, Brady M, Smith S. Segmentation of brain MR images through a hidden Markov random field model and the expectation-maximization algorithm. IEEE Trans Med Imaging. 2001 Jan;20(1):45–57. doi:[**10.1109/42.906424**](https://doi.org/10.1109/42.906424).

18. Behzadi Y, Restom K, Liau J, Liu TT. A component based noise correction method (CompCor) for BOLD and perfusion based fMRI. Neuroimage. 2007 Aug 1;37(1):90–101. doi:[**10.1016/j.neuroimage.2007.04.042**](https://doi.org/10.1016/j.neuroimage.2007.04.042).

19. Power JD, Mitra A, Laumann TO, Snyder AZ, Schlaggar BL, Petersen SE. Methods to detect, characterize, and remove motion artifact in resting state fMRI. Neuroimage. 2013 Aug 29;84:320–41. doi:[**10.1016/j.neuroimage.2013.08.048**](https://doi.org/10.1016/j.neuroimage.2013.08.048).

20. Pruim RHR, Mennes M, van Rooij D, Llera A, Buitelaar JK, Beckmann CF. ICA-AROMA: A robust ICA-based strategy for removing motion artifacts from fMRI data. Neuroimage. 2015 May 15;112:267–77. doi:[**10.1016/j.neuroimage.2015.02.064**](https://doi.org/10.1016/j.neuroimage.2015.02.064).

21. Klein A, Ghosh SS, Bao FS, Giard J, Häme Y, Stavsky E, et al. Mindboggling morphometry of human brains. PLoS Comput Biol 13(2): e1005350. 2017. doi:[**10.1371/journal.pcbi.1005350**](https://doi.org/10.1371/journal.pcbi.1005350).

22. Abraham A, Pedregosa F, Eickenberg M, Gervais P, Mueller A, Kossaifi J, Gramfort A, Thirion B, Varoquaux G. Machine learning for neuroimaging with scikit-learn. Front in Neuroinf 8:14. 2014. doi:[**10.3389/fninf.2014.00014**](https://doi.org/10.3389/fninf.2014.00014).
